# How and when empowering leadership influences job performance: A dual-mediator model

**DOI:** 10.1371/journal.pone.0332545

**Published:** 2026-02-13

**Authors:** Hong Liang, Hooi Hooi Lean, Asokan Vasudevan

**Affiliations:** 1 School of Management, Universiti Sains Malaysia, Pulau Pinang, Malaysia,; 2 Department of Science, Technology, and International Projects, Ho Chi Minh City University of Economics and Finance (UEF), Ho Chi Minh City, Vietnam; 3 Faculty of Business and Communications, INTI International University, Nilai, Malaysia; Guangxi Normal University, CHINA

## Abstract

Based on the Social Exchange Theory and Self-Determination Theory, this study examines the impact of empowering leadership on employee job performance through the underlying mechanisms of structural empowerment and team cohesion. Survey data was collected using self-administrated questionnaires from 483 secondary school teachers in Hong Kong. Partial Least Square-Structural Equation Modeling technique was used to examine the proposed hypotheses of the study. Survey data from a cross-sectional sample of 483 secondary school teachers in Hong Kong was used to test the parallel mediation model. The results of structural equation modeling indicate that empowering leadership, structural empowerment, and team cohesion serve as direct motivating factors of job performance. Additionally, empowering leadership was found to indirectly enhance job performance via the mediation of structural empowerment and team cohesion. This research addresses a knowledge gap in understanding the mechanisms through which empowering leadership effectively improves work outcomes, particularly in the school setting. Leaders of educational institutions may consider increasing teachers’ job autonomy as a strategy to enhance workplace performance.

## 1.0. Introduction

In today’s business era, accelerated globalization and constant changes make firms face high demand to enhance their performance and boost competitiveness [1]. This puts employees under pressure to use their capabilities and competencies to benefit their organization in dealing with a changeable business environment. Therefore, organizations need employees who are emotionally connected to their work and accept every challenge for the prosperity and progress of the organization. Leaders believe that fulfilled, skilled, engaged, and loyal employees are the most important asset of the organization and hence they are working towards improving the well-being and job satisfaction of their employees [2]. To overcome these organizational challenges, organizations should build potential and encouraging relationships with their employees and ensure their work efficiency and performance. A high level of job performance helps organizations establish an environment with dynamic change where organizations can survive and achieve their desired goals [3].

Given the growing recognition that leadership can be studied at the group level, it is clear that followers are influenced by a shared leader [4]. This has led to increasing interest in leadership approaches that align with the rapidly changing business environment, enabling organizations to better navigate sustained uncertainties [5]. Many organizations are working towards structural changes by leaving behind the traditional way of organizational management and introducing an empowering leadership style where leaders give more power in decision-making and take more responsibility for the work of their subordinates with the aim of improving the productivity of the organization. Empowering leadership, in particular, has emerged as a critical intangible asset for organizations and a key predictor of competitive advantage in today’s fast-paced global landscape [6]. Amundsen et al. [7] identified three key components of empowering leadership: shared power, motivational support, and developmental support. Empowering leadership creates an environment conducive to the development and autonomy of subordinates, which is considered to be a factor that can indirectly promote employee engagement and task performance by promoting the development of individual potential and motivation [8]. Empowering leadership enables subordinates to recognize their potential and limitations and gain competence in professional development, which is considered to be a factor that improves job performance. According to social exchange theory (SET), empowering leadership is seen as leaders treating employees positively and supporting their work by giving them autonomy and trust in their responsibilities. Employees will strive to repay leaders by improving their performance. This study shows that organizational support promotes individuals to have positive reciprocity tendencies to help the organization.”

It has been proposed that structural empowerment is an important predictor of job performance [9]. empowering leaders can cultivate structural empowerment, which provides employees with greater job autonomy and organizational support [10]. Such support inspires a sense of security and motivation, enabling employees to work harder, handle tasks more effectively, and deliver better results for the organization. The literature suggests that the increase in perceived access to workplace empowerment structures will predict higher performance [11]. Accordingly, empowering work conditions may enhance job performance by stimulating employees’ intrinsic and extrinsic motivation. At the same time, researchers have found a strong relationship between team cohesiveness and job performance [12]. Team cohesion, defined as a dynamic process that reflects a group’s desire to unite and work toward shared goals or meet the emotional needs of its members, is key to team success. In the meta-analysis study that analyzed the relationship between group cohesion and performance, Evans et al. [13] found that cohesive groups outperformed non-cohesive groups by approximately 18 percent. Leadership studies have shown that team cohesion improves group processes, such as team member coordination, communication, mutual support, participation and effort [14]. However, the dual mediation effects of structural empowerment and team cohesion between empowering leadership and job performance remain underexplored.

The concept of empowering leadership and employees’ job performance has not been researched in the context of secondary school of Hong Kong till now. Therefore, this study found a research area gap. Previous studies mostly focused on commercial industries [15], banking sectors [16], hospitality [17], pharmaceutical [18], manufacturing [19], and construction [20] but ignored academic institutions. Thus, this study found a population gap. Moreover, In the previous study, goal clarity and self-efficacy mediate partially the relationship between empowering leadership and job performance [21]. So, this present study can analyze this relationship with the presence of further mediating variables such as knowledge sharing, structural empowerment, team cohesion, and psychological empowerment. Therefore, this study are conducting this research to further enhance the existing literature gap. As compared to previous studies, this study have employed a large sample size to get more generalized results. Consequently, this study filled this methodological research gap. The association between empowering leadership and employees’ job performance is a unique concept that is mostly ignored as mentioned above and requires more investigation. Hence, this study found a conceptual research gap. This study is based on the academic staff perspective which is not found in previous studies. Thus, this study found a perspective research gap.

Although empowering leadership has been widely studied, significant gaps remain in understanding its influence on job performance within the education sector. Most previous research has focused on commercial industries, leaving limited evidence from educational contexts such as secondary schools in Hong Kong. Furthermore, prior studies have mainly examined simple mediators like goal clarity or self-efficacy, overlooking the roles of structural empowerment and team cohesion in explaining how empowering leadership enhances performance. Methodologically, small and non-representative samples and single-mediator models have limited the robustness of past findings. Therefore, this study aims to address these contextual, conceptual, and methodological gaps by investigating empowering leadership among Hong Kong secondary school teachers through a parallel mediation model incorporating structural empowerment and team cohesion.

To address the gaps mentioned above, this study aimed to investigate the direct and indirect impact of empowering leadership on job performance among Hong Kong secondary school teachers, with structural empowerment and team cohesion serving as parallel mediators. The findings are expected to contribute to the empowering leadership literature as well as to educational management in Hong Kong. The following section of this paper presents the study’s theoretical foundation and hypotheses development. Subsequently, the research methodology and analysis results are detailed. The paper concludes by discussing the study’s findings, implications, limitations, and recommendations for future research.

This study addresses three major research gaps—contextual, conceptual, and methodological—within the empowering leadership and job performance literature. First, the contextual gap is bridged by investigating this relationship in the education sector, particularly among secondary school teachers in Hong Kong, a context that has been largely overlooked in previous studies. Earlier research has predominantly examined empowering leadership within commercial industries [15], banking sectors [16], hospitality [17], pharmaceutical [18], manufacturing [19], and construction [20], leaving educational institutions underexplored. Second, the study fills a conceptual gap by expanding the theoretical understanding of how and why empowering leadership influences job performance through the inclusion of structural empowerment and team cohesion as parallel mediators [77]. While previous studies have focused primarily on mediators such as goal clarity and self-efficacy, this research integrates social exchange theory and self-determination theory to explain both the individual and collective mechanisms that drive employee performance. Third, the study addresses a methodological gap by employing a larger and more representative sample of participants from the education sector, thereby improving the generalizability and validity of findings. Furthermore, it adopts a parallel mediation analytical approach, which provides a more rigorous and comprehensive examination of the indirect effects between variables compared to earlier single-mediator models.

## 2.0. Theoretical foundation and hypotheses development

### 2.1. Social Exchange Theory (SET)

Widely used in the fields of management and sociology, the SET explains behavior based on principles from economics and behavioral psychology. According to Cook et al. [22], it aims to explain behavior rather than merely describe it, focusing on interactions between two or more individuals and how these interactions reinforce each other’s behavior. One key reason for the SET’s appeal is its simplicity and applicability across a wide range of social contexts. In this study, the concept of social exchange encompasses both content and process. Content refers to the resources exchanged between supervisors and subordinates, while process involves “how” these exchanges take place—specifically, how supervisors and subordinates mutually benefit from the interaction.

In the existing literature, SET is mainly used to study three aspects: the initiation of reciprocal behavior (e.g., providing information support), the reciprocal process (e.g., the formation of a bilateral relationship), and the reciprocal response (e.g., attitudinal and behavioral outcomes). In addition, positive reciprocal beliefs have been studied as a moderator in these contexts [23]. First, understanding SET is crucial to understanding how information exchange acts as a priming action that triggers reciprocal exchanges between employees and leaders. For example, when employees find the information shared by leaders valuable, credible, and vivid, this triggers positive emotions such as confidence and excitement, motivating them to invest energy in establishing an emotional connection with their leaders. Second, employee engagement can be viewed as a social exchange (reciprocal process) between employees and leaders. This reciprocal process can indirectly affect job performance by creating a sense of community and emotional satisfaction [24]. Third, as discussed in Cropanzano et al. [23] review of SET, positive reciprocal beliefs may act as a potential moderator that affects individual attitudes and behaviors. Relationship orientation is a positive reciprocal belief rooted in Chinese culture that regulates individual attitudes and behaviors by fostering trust, reciprocity, emotional connection, and enhancing persuasion [25].

The revised SET explains that interpersonal exchange is based on six rules: reciprocity, rationality, altruism, group gain, status consistency, and competition. Rationality refers to the logic behind personal behaviour based on values and beliefs. A leader’s values and beliefs influence followers’ perceptions. In order to overcome the shortcomings of SET, Sari et al. [26] utilized Weber’s theory of rationality to explain how these factors interact with employees’ values and beliefs to influence their perceptions. Altruism refers to doing something for the benefit of others [27]. Group benefits refer to the benefits gained by society rather than individuals; in turn, individuals benefit from the group. Status consistency suggests that belonging to a certain group, such as race or gender, can benefit individuals and influence their perceptions. Competition rules are the opposite of altruism; that is, individuals act against others regardless of the harm they may cause themselves. Using this revised SET framework, identifying the factors that influence an employee’s job performance may be easier and more understandable.

### 2.2. Self-Determination Theory (SDT)

Self-Determination Theory (SDT) has evolved over four decades into a widely accepted macro-theory of human motivation, personality, and well-being, focusing on the factors that support or hinder human potential and self-directed behavior [28]. SDT specifically suggests that both employees’ performance and their well-being are affected by the type of motivation they have for their job activities [29]. SDT therefore differentiates types of motivation and maintains that different types of motivation have functionally different catalyzers, concomitants, and consequences.

The core rationale of SDT is that an individual has three innate psychological needs and that the degree of their satisfaction is a prerequisite for boosting positive behavior at work and well-being [30]. Specifically, autonomy concerns the need for a sense of the volition and self-endorsement of behavior [31]. Relatedness refers to the requirement of experiencing a close connection with other people [32]. The need for competence refers to an employee feeling confident when handling challenging tasks [33]. Although the strength of each need may vary individually, when basic psychological needs (BPN) are adequately satisfied by environmental conditions, individuals experience psychological growth, internalization and well-being. In the context of work, BPN is determined by the structural or social aspects of the work environment [34]. For instance, a work climate that promotes growth and goal achievement has been shown to facilitate employees’ BPN at work [35].

### 2.3. Empowering leadership and job performance

Numerous studies analytically supported the impact of empowering leadership on workers’ job performance. Employees’ efficacy can be increased if their leaders use an empowering leadership style. Empowering leadership stems from the belief that employees who have more opportunities for self-guidance will exhibit better results [36]. Empowering leadership emphasizes the sharing of power between leaders and subordinates, and emphasizes the ability of leaders to enhance subordinates’ sense of meaning, ability, self-determination, and influence [37]. Some empirical studies support the view that empowering leadership improved employee job performance [38]. Empowering leadership is also considered an important driving factor for organizational effectiveness [39].

By adopting an empowering leadership style, leaders expect employees to feel responsible for their work and know how to complete their work successfully. Empowering leaders encourages subordinates to rise to high levels of performance in their jobs; indeed, job performance occurs when employees work with high levels of energy and strongly identify with their jobs [40]. Kahn [41] believed that employees perform better when they feel that the boundaries of their jobs are clear and defined. In addition, employees perform better when empowering leaders recognize their self-worth and job roles, such as when leaders share information and knowledge to make employees more effective [42].

Empowering leaders provides teachers with autonomy and opportunities, which eliminates bureaucratic constraints, promotes information dissemination, and fosters trust and communication within the organization [43]. This delegation of authority helps teachers understand the broader significance and impact of their work, consequently building their sense of self-determination, purpose, competence, and influence [44]. When teachers feel psychologically empowered—through meaning, involvement, and autonomy—their confidence, engagement and loyalty increase, motivating them to take responsibility and contribute more effectively [45]. Therefore, empowering leadership can improve both task and situational performance.

Integrating SDT and SET provides a comprehensive explanation on why empowering leadership leads to a better job performance. SDT explains the internal motivational mechanism, whereas empowerment satisfies psychological needs and fosters intrinsic motivation. SET explains the relational mechanism, whereas empowerment creates a sense of obligation and mutual trust that encourages employees to reciprocate with higher performance. Together, these theories demonstrate that empowering leadership not only motivates employees internally but also strengthens social exchanges, both jointly improve job performance. Based on this discussion, the following hypothesis is proposed:

H1: Empowering leadership is positively related to job performance.

### 2.4. Empowering leadership and structural empowerment

Empowerment implies a range of management practices (e.g., sharing authority, resources, information) that directly affect work outcomes (e.g., quality, productivity, customer satisfaction). It also indirectly affects them by influencing employee cognitions (e.g., self-efficacy, motivation). Empowerment is a management practice that plays a key role in the career development of employees [46]. Laschinger et al. [47] tested a model that links structural empowerment to six domains of work life that are prerequisites for job engagement or low levels of burnout. Research suggests that empowering workplaces can increase employees’ levels of control over their work, increase workload, increase rewards and recognition for employees’ contributions to achieving organizational goals, improve working relationships between colleagues and management, and increase congruence between personal and organizational values [48].

According to Kanter [49], employees are empowered when they have access to necessary information, learning and development opportunities, support, and resources at work. Such a workplace structure should include access to information about policies, organizational results, and organizational changes through an open communication system. Opportunities for learning and development are another dimension that enables employees to advance professionally in an organization. Support includes receiving feedback and guidance from subordinates, peers, and supervisors. Resources refer to access to materials and equipment, time, and financial resources necessary to achieve organizational goals [50]. Therefore, according to social exchange theory, when leaders provide these social structures for employees, they feel empowered and are allowed to complete their work in a meaningful way.

From the perspective of SDT, empowering leadership enhances structural empowerment by embedding autonomy, competence, and relatedness into formal organizational structures through delegated authority, shared decision-making, and clear roles. From the perspective of SET, empowering leadership strengthens structural empowerment by fostering reciprocal relationships, where employees respond to leaders’ trust and support by actively engaging with and sustaining empowering organizational structures.

Empowering leaders delegate authority, allowing teachers the freedom to make decisions and take action within their roles, while providing proper guidelines to ensure teachers understand their responsibilities [51]. Empowering leaders also offers teachers the resources, information, and support needed for success, facilitating informed decision-making. By investing in skill development through training and mentoring, these leaders help teachers take on more complex tasks and develop a sense of competence [52]. Clear expectations and goals further establish a structured framework that allows teachers to work independently, with full confidence and appreciation towards their job scope. In line with these arguments, we hypothesize that:

H2: Empowering leadership is positively related to structural empowerment.

### 2.5. Empowering leadership and team cohesion

Empowering leadership has a positive impact on team cohesion by enhancing team members’ intrinsic motivation and strengthening reciprocal interpersonal relationships within the team. Team cohesion is considered as the primary aspect of human resource management in project-intensive organizations [53]. Team cohesion is essentially about transforming a group of employees from different parts of an organization into a cohesive unit. Sohmen [54] emphasized that teams are close-knit groups and close relationships between participants are essential to achieving organization goals. Goal setting, clear roles, interpersonal relationships, and problem-solving are unique approaches to team building [55]. Interpersonal relationships help mitigate conflicts and disputes among project team members [56]. Problem-solving helps identify and resolve major issues with team members’ tasks.

Based on social exchange theory, we argue that empowering leaders brings team members together with the ultimate goal of achieving a common goal in mind while enhancing team cohesion and creating an environment of mutual respect. Empowering leaders generate a sense of trust in team members’ ability to achieve common goals and treat each team member equally. As a result, team members tend to feel satisfied with their roles and are more engaged in their work in return [57].

Team cohesion is strongly influenced by leadership behaviors, such as delegating power to teachers, expressing trust in them, and validating the importance of their work [58]. When teachers feel connected and empowered through these actions, they develop stronger cohesion and identity. According to Tang et al. [59], empowering leadership is closely tied to intrinsic motivation and team efficacy, both of which are crucial psychological drivers of team cohesion. In addition, this type of leadership tends to boost team confidence, foster a shared mindset, and encourage risk-taking, ultimately enhancing team trust and cohesion [60,61]. Empowering leaders also leads by example, guiding teachers in skill-building and motivating them to take on tasks, which further enhances their cohesion.

From the perspective of SDT, empowering leadership promotes team cohesion by satisfying team members’ basic psychological needs for autonomy, competence, and relatedness in a collective context. At the same time, empowering leadership emphasizes collaboration, open communication, and mutual respect, thereby strengthening feelings of relatedness among team members. From the perspective of SET, empowering leadership enhances team cohesion through reciprocal exchanges based on trust and mutual obligation. Team members perceive these empowering behaviors as valuable socio-emotional resources and feel obligated to reciprocate not only toward the leader but also toward their teammates. This reciprocity encourages behaviors such as mutual assistance, knowledge sharing, and conflict resolution, which strengthen interpersonal ties and collective commitment. Therefore, the following hypothesis is proposed:

H3: Empowering leadership is positively related to team cohesion.

### 2.6. Structural empowerment and job performance

Structural empowerment has a positive impact on job performance by providing employees with access to resources, information, support, and opportunities that enhance motivation and strengthen reciprocal work relationships. Structural empowerment has been linked to various work-related outcomes, such as job strain, mental fatigue, and stress [62]. It has been suggested that structural empowerment is an important predictor of job performance [63]. For example, the literature suggests that an increase in perceived workplace empowerment structure predicts higher performance [64].

According to SET, an empowering environment enables employees to face work-related challenges. The benefits of structural empowerment can be manifested in improved employees’ attitudes and progress towards meeting organizational goals, which promotes organizations’ success [65]. By providing empowering structures such as participation mechanisms, access to resources, and opportunities for growth, the organization signals trust, support, and respect for employees. In response, employees feel obligated to reciprocate through positive work behaviors, including higher task performance, greater commitment, and extra-role contributions.

From the perspective of SDT, structural empowerment improves job performance by creating organizational conditions that satisfy employees’ basic psychological needs for autonomy, competence, and relatedness. When employees have access to information, decision-making authority, and discretion over their work, they experience greater autonomy embedded within organizational structures. Access to training, feedback, and necessary resources enhances employees’ sense of competence, enabling them to perform tasks more effectively and confidently.

Structural empowerment involves mobilizing people and resources to achieve organizational goals, facilitated by access to relevant information, support, opportunities, and resources. First, access to information includes awareness of organizational changes, policies, and the technical knowledge required to fulfill responsibilities. Next, opportunities for development emerge when teachers are able to learn, grow, and advance within the organization by building their skills and competencies [66]. Third, support is derived from feedback and guidance from subordinates, colleagues, and superiors. In terms of resources, providing teachers with good working conditions aids them in achieving their work objectives, which stimulates both internal and external motivation [67]. Altogether, when teachers are structurally empowered via access to information, support, development opportunities, and necessary resources, their basic needs for autonomy, competence, and relatedness are fulfilled. As such, they are motivated to work harder and attain positive performance outcomes. Based on this discussion, this study predicts that:

H4: Structural empowerment is positively related to job performance.

### 2.7. Team cohesion and job performance

Team cohesion has a positive impact on job performance by enhancing employees’ motivation, coordination, and willingness to contribute to collective goals. Research shows that team cohesion is critical to team performance when teams work in highly stressful and task-oriented environments [68]. This explains why cohesion is a key factor for success [69]. Team literature shows that cohesion has a positive impact on all dimensions of team effectiveness, namely performance, satisfaction, and viability. Cohesive teams tend to perform better and show satisfied members who are willing to stay in the team [70].

Team cohesion creates a favorable working environment, enhances team members’ cooperation and goal-oriented efforts, and reduces role ambiguity, thus ensuring the success of work [71]. According to SET, team cohesion promotes good working relationships between school administrators and teachers, resulting in team synergy. Therefore, teachers value a favorable working atmosphere and are committed to and motivated to achieve project goals [72].

Supporting this, Elron [73] found that managing team cohesion improves team performance, particularly in decision-making and strategy implementation, as well as overall vision and goal alignment. A strong foundation of interpersonal connections and shared values enables cohesive teams to operate flexibly and productively [74]. As a result, cohesive teams are more united and committed to success than less cohesive ones, as the teachers better utilize their strengths by understanding their teammates and working together toward common goals.

From the perspective of SDT, team cohesion improves job performance by fulfilling employees’ basic psychological needs for relatedness, autonomy, and competence within the team context. At the same time, cooperation and mutual support within cohesive teams enable knowledge sharing and skill development, strengthening employees’ sense of competence. When these psychological needs are satisfied, employees are more intrinsically motivated to engage fully in their tasks, coordinate effectively with teammates, and maintain high levels of effort, resulting in improved job performance.

From the perspective of SET, in cohesive teams, individuals engage in frequent positive exchanges, such as helping behaviors, information sharing, and emotional support. These exchanges create mutual obligations and expectations of reciprocity, motivating employees to contribute more to the team’s success. Based on this, the following hypothesis is proposed:

H5: Team cohesion is positively related to job performance.

### 2.8. The mediating roles of structural empowerment and team cohesion

#### 2.8.1. The mediating role of structural empowerment.

When employees experience structural empowerment, they feel more autonomous in their work, capable of handling tasks effectively, and supported by the organization. Boamah et al. [75] found that structural empowerment, along with psychological capital, was positively associated with job performance. Furthermore, Laschinger et al. [76] showed that structural empowerment was associated with greater employee productivity and work engagement. Prior empirical evidence Saks et al. [77] suggests that factors that constitute structural empowerment (e.g., performance feedback, development opportunities, organizational and social support) can foster job performance.

SET provides a theoretical basis for explaining the relationship between leadership style and acceptance of structural empowerment and job performance. The theory explains social exchange as a process of negotiated exchange between two parties, which involves reciprocity. That is, when a relationship is established between a leader and an employee, certain reciprocal obligations arise, such as psychological meaning, security, or availability when the leader demonstrates genuine personal recognition or supportive leadership [78]. In addition, when an organization provides a resource-rich work environment and job resources (such as support, information, or feedback), employees may feel compelled to reciprocate with high levels of engagement [79]. Although both transformational and empowering leadership may contribute to job performance [80], we believe that acceptance of empowering leadership is primarily related to job performance because structural empowerment is enhanced.

Structural empowerment strengthens this process by institutionalizing access to resources, information, and opportunities, making it easier for employees to reciprocate effectively. From the perspective of SDT, empowering leaders enhance structural empowerment by providing employees with access to resources, information, decision-making authority, and opportunities for growth. These organizational structures satisfy employees’ basic psychological needs for autonomy, competence, and relatedness. Thus, structural empowerment acts as a conduit through which the motivational benefits of empowering leadership are realized in tangible performance outcomes.

According to Lewis et al. [81] conceptual model, systemic power factors influence the acquisition of work-related empowerment structures, which in turn have personal impacts on employees and their organizational effectiveness. Systemic power factors include formal power characteristics (job definition, discretion [flexibility], recognition [visibility], and relevance [centrality]) or informal power characteristics (internal organizational connections, alliances with sponsors, colleagues, subordinates, cross-functional groups, and external organizational connections) [82]. These characteristics interact with resource and information access, ultimately leading to increased self-efficacy, high motivation, increased organizational commitment, reduced burnout levels, increased autonomy, reduced occupational stress, increased job satisfaction, and overall positive organizational and personal well-being [83]. This phenomenon, called structural empowerment, allows employees to perform their job duties effectively by enhancing their capabilities, clarifying roles and expectations, cultivating autonomy, and supporting decision-making [84]. Ultimately, empowering individuals through defined structures fosters a sense of ownership and responsibility, improving their motivation, satisfaction, and performance at work. Thus, in line with prior theoretical and empirical work, the following hypothesis is formulated:

H6: Structural empowerment mediates the relationship between empowering leadership and job performance.

#### 2.8.2. The mediating role of team cohesion.

Empowering leadership enhances cohesion, and cohesive teams are more effective at translating motivation and cooperation into improved job performance. Several meta-analyses have positively correlated cohesion with performance [85]. Team cohesion is believed to be able to effectively maintain good intra-team relationships [86]. Such teams have a common vision and motivation to achieve organizational goals. Cohesive teams show more creativity and higher performance than less cohesive teams [87]. Previous research supports a consistent relationship between cohesion and performance [88].

According to self-determination theory, members of a cohesive team are willing to put in more effort in performing tasks because completing tasks that team members enjoy brings them intrinsic pleasure. Strong cohesion has also been shown to accelerate individual efforts and perseverance to achieve team goals, thereby making team actions harmonious and consistent [89].

From the perspective of SET, empowering leadership enhances team cohesion by creating a climate of trust, support, and mutual obligation. When leaders empower team members, they signal respect and confidence, which encourages reciprocal behaviors. Team members respond by helping each other, sharing resources, and coordinating effectively, strengthening interpersonal bonds and collective commitment. This cohesion, in turn, facilitates collaboration and reduces conflict, enabling higher task efficiency and performance.

In addition, from the perspective of the task dimension, cohesion affects team processes and outputs, affecting the decision-making process and response speed of each member [90]. In the process of fighting and completing tasks together, team members begin to develop camaraderie and a sense of belonging to the team, which further contributes to cohesion and performance [91]. According to Oh et al. [92], strong cohesion encourages sharing of responsibility for failure and allows members to bear the negative consequences of destructive events.

**Fig 1:**
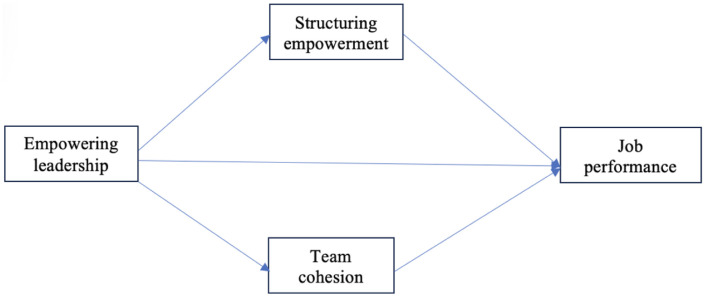
Depicts the conceptual framework of this study.

Previous research on team leadership has shown that when leaders and followers are compatible, the effectiveness of social interactions will increase, thereby promoting the development of social identity, team cohesion between leaders and followers, and desired team behaviors (Fig 1) [93]. Shared responsibility also creates an environment in which team members’ sense of ownership and commitment to achieving team goals increase, resulting in creative ideas [94]. Given the link between team cohesion and job performance, we echo the call of Ahmed et al. [21], who emphasized the need for a mediating mechanism of team cohesion between empowering leadership and job performance. For example, if the members of the teaching team have strong cohesion, they can keep abreast of the latest market trends and complete their job performance, so the path between team cohesion and job performance is confirmed [95]. However, there is less understanding of the underlying mechanism of team cohesion, therefore, the following hypotheses are proposed:

H7: Team cohesion mediates the relationship between empowering leadership and job performance.

## 3.0. Methods

### 3.1. Sample and data collection

The research sample consisted of academic staff from four secondary schools in Hong Kong. The questionnaire used in this study was carefully adapted to Hong Kong’s educational context to ensure cultural relevance, clarity, and validity of the measurement items. All survey instruments were sourced from established and validated scales in prior leadership and organizational behavior research. The initial version of the questionnaire underwent expert review by educational management scholars and experienced secondary school teachers in Hong Kong to assess the appropriateness, cultural sensitivity, and clarity of each item. Respondents were required to meet three inclusion criteria: (i) currently registered as a secondary school teacher in Hong Kong, (ii) possesses at least a Bachelor’s degree qualification, and (iii) has a minimum of one year of teaching experience. Initially, we contacted the schools via email to secure their human resource managers’ approval to participate in the study. Once they agreed, online questionnaires were distributed to the teachers through Google Forms. The cover letter of the form explained that participation was voluntary and that all responses would be confidential and used solely for research purposes.

All procedures involving human participants in this study were in accordance with the ethical standards of the Faculty Research & Ethics Panel of INTI International University (INTI/UEC/2024/024), and the 1964 Helsinki Declaration and its later amendments. Before data collection, participants were informed about the study’s purpose, their voluntary participation, and the confidentiality of their responses through an electronic consent form. Ethical approval was obtained from the university ethics committee, ensuring the study complied with international standards for human research involving respect, beneficence, and justice. Participation was voluntary and anonymous, based on written informed consent and the right to withdraw participation at any time. Further, the researcher collected the questionnaires immediately after completion.

A total of 600 questionnaires were distributed, of which 483 were completed and deemed valid for analysis (i.e., 80.5% response rate). All respondents are Hong Kong nationals. As shown in Table 1, 48.9% of respondents were male and 51.1% were female, indicating a relatively balanced gender distribution. The majority were between 21 and 30 years old (58%), 22.6% were 31–40 years old, and 18.6% were 41–50 years old. In terms of educational background, 59.2% held a Bachelor’s degree, 38.9% held a Master’s degree, and 1.9% held a doctoral degree. Lastly, most of the respondents had four to five years of teaching experience (47.2%), 22.6% had six to eight years of experience, and 19% had over nine years of experience.

**Table 1:** Demographic characteristics of respondents.

Characteristics	Frequency	Percentages (%)
Gender		
Male	236	48.9%
Female	247	51.1%
Age		
21-30	280	58%
31-40	109	22.6%
41-50	90	18.6%
Above 51	4	0.8%
Educational level		
Bachelor degree	286	59.2%
Masters’ degree	188	38.9%
Ph.D degree	9	1.9%
Monthly income		
HKD 10,000–20,000	297	61.5%
HKD 21,000–30,000	179	37.1%
HKD 31,000–40,000	7	1.4%
Above HKD 41,000		
Nationality		
Hong Kong	483	100%
US		
UK		
Work experience		
1–3 years	54	11.2%
4–5 years	228	47.2%
6–8 years	109	22.6%
Above 9 years	92	19%
Total	483	80.5%

### 3.2. Measures

The questionnaire was administered in English, one of Hong Kong’s official languages. All four variables’ measurement items were adapted from previous studies and rated on a five-point Likert scale, ranging from 1 (strongly disagree) to 5 (strongly agree). First, empowering leadership was assessed using six dimensions, including delegation of authority, accountability, self-directed decision-making, information sharing, skill development, and coaching for innovative performance adapted from Konczak et al. [62]. A sample item is “My manager focuses on corrective action rather than placing blame when I make a mistake.” Cronbach’s alpha values for the scale ranged from 0.82 to 0.97. Second, structural empowerment was measured using three subscales: opportunity (e.g., “gain new skills”), support (e.g., “problem-solving help”), and resources (e.g., “time to accomplish job”), based on the nine-item scale developed by Laschinger et al. [96]. Cronbach’s alpha values for the scale ranged from 0.71 to 0.95. Third, team cohesion was measured using eight items adapted from Short et al. [97], with Cronbach’s alpha values ranging from 0.67 to 0.85. An example item is “The team I belonged to was a close one.” Finally, job performance was measured using four items adopted from Yilmaz et al. [98]. Sample items included “I complete my tasks on time” and “I respond quickly when problems arise.” The Cronbach’s alpha for this scale was 0.82.

## 4.0. Analysis and results

### 4.1. Assessment of measurement model

PLS-SEM was chosen over CB-SEM in this study because it is more suitable for complex models that include formative constructs, multiple mediators, and a predictive research orientation. Unlike CB-SEM, which focuses on theory confirmation and requires strict assumptions of normal data distribution, PLS-SEM emphasizes maximizing explained variance in the dependent variables, making it ideal for exploratory and prediction-oriented studies. Additionally, PLS-SEM can effectively handle smaller to moderate sample sizes and complex path relationships without compromising statistical power. Given that this study aims to predict job performance based on empowering leadership and its mediating mechanisms, PLS-SEM provides a more flexible and robust analytical approach than CB-SEM. The collected survey data was analyzed using partial least squares structural equation modeling (PLS-SEM). Following Anderson et al. [99] two-step approach, we first evaluated the measurement model to ensure construct validity and reliability before proceeding to test the structural model. All indicators with a factor loading greater than 0.5 are considered for further analysis. The reflective measurement model analysis included assessments of factor loadings, average variance extracted (AVE), and composite reliability (CR). According to the guidelines of Hair et al. [100] and Ramayah et al. [101], CR should be ≥ 0.7, AVE should be ≥ 0.5, and factor loadings should be greater than 0.5. As shown in Table 2, both AVE and CR values exceeded the thresholds of 0.5 and 0.7, respectively. Thus, the first-order constructs’ internal consistency reliability and convergent validity were confirmed. Since two second-order constructs—empowering leadership and structural empowerment—were present in the model, the validity and reliability of these second-order constructs were also assessed, as indicated in Table 3. The second-order measures were found to be demonstrate reliability and convergent validity as well.

**Table 2:** Measurement model for first-order constructs (reflective).

First-order construct	Items	Loadings	CR	AVE
Delegation of authority	EL1	0.936	0.890	0.604
	EL2	0.697		
	EL3	0.916		
Accountability	EL4	0.883	0.905	0.760
	EL5	0.856		
	EL6	0.875		
Self-directed decision making	EL7	0.802	0.887	0.725
	EL8	0.915		
	EL9	0.832		
Information sharing	EL10	0.873	0.852	0.742
	EL11	0.849		
Skill development	EL12	0.871	0.852	0.742
	EL14	0.852		
Coaching for innovative performance	EL15	0.744	0.820	0.604
	EL16	0.854		
	EL17	0.727		
Team cohesion	TC1	0.724	0.935	0.645
	TC2	0.584		
	TC3	0.851		
	TC4	0.877		
	TC5	0.889		
	TC6	0.817		
	TC7	0.883		
	TC8	0.750		
Opportunity	SE2	0.761	0.885	0.721
	SE3	0.742		
Support	SE4	0.944	0.851	0.660
	SE5	0.703		
	SE6	0.771		
Resource	SE7	0.789	0.834	0.627
	SE8	0.841		
	SE9	0.743		
Job performance	JP1	0.513	0.821	0.541
	JP2	0.732		
	JP3	0.948		
	JP4	0.805		

Note. (a): EL = empowering leadership; SE = structural empowerment; TC = team cohesion; JP = job performance.

(b): EL13 and SE1 were deleted due to low loadings.

**Table 3:** Measurement model of second-order constructs (formative).

Second order constructs	Indicator	Loadings	CR	AVE
Empowering leadership	DA	0.794	0.901	0.604
	ACC	0.879		
	SDDM	0.762		
	IS	0.670		
	SD	0.744		
	CIP	0.800		
Structural empowerment	OPP	0.799	0.860	0.673
	SP	0.782		
	RE	0.877		

Note: Acc = Accountability; CIP = Coaching for innovative performance; DA = Delegation of authority; IS = Information sharing; OPP = Opportunity; RE = Resource; SDDM = Self-directed decision making; SD = Skill development; SP = Support.

To evaluate discriminant validity, the heterotrait-monotrait (HTMT) criterion was employed (see Table 4), whereby values should not exceed the threshold of 0.85. In this study, all HTMT values were below 0.85, satisfactorily confirming discriminant validity for all the constructs.

**Table 4:** Discriminant validity (HTMT criterion).

Constructs	EL	JP	SE	TC
1. Empowering Leadership				
2. Job Performance	0.827			
3. Structural Empowerment	0.838	0.743		
4. Team Cohesion	0.716	0.612	0.430	

Note. EL = empowering leadership; SE = structural empowerment; TC = team cohesion; JP = job performance.

### 4.2. Common method variance

The issue of potential collinearity was assessed using Variance Inflation Factor (VIF) values, based on a common method variance test. According to Kock et al. [102] and Kock [103], a VIF score of 3.30 or below is required to eliminate concerns of bias. In this study, all VIF values were found to be below 3.30 (see Table 5), indicating that the dataset did not suffer from common method bias.

**Table 5:** Full collinearity testing.

Variable	EL	JP	SE	TC
VIF	1.529	2.839	2.254	1.790

Note: EL = empowering leadership; SE = structural empowerment; TC = team cohesion; JP = job performance.

To minimize common method bias (CMB) beyond the statistical assessment using variance inflation factor (VIF), several procedural remedies were implemented during the research design and data collection stages. First, anonymity and confidentiality were assured to all respondents, emphasizing that their responses would be used solely for academic purposes and that no identifying information would be disclosed. This approach reduced social desirability bias and encouraged participants to provide honest and unbiased answers. Second, psychological separation was introduced between predictor and criterion variables by organizing the questionnaire into distinct sections and varying the response formats, which helped reduce respondents’ tendency to infer relationships among variables. Third, clear and neutral wording was used throughout the questionnaire to avoid leading questions and reduce ambiguity. Lastly, participants were informed that there were no right or wrong answers, reinforcing that their genuine perceptions were valuable to the study. Together, these procedural measures helped mitigate the risk of common method variance and improved the overall reliability and validity of the collected data.

### 4.3. Assessment of structural model

The structural model was analyzed to test the proposed hypotheses using bootstrapping with 10,000 subsamples [104], the results of which are presented in Table 6. Empowering leadership was found to have a significant positive influence on job performance (β = 0.855, p < 0.05), structural empowerment (β = 0.655; p < 0.05), and team cohesion (β = 0.780; p < 0.05). Therefore, H1, H2, and H3 were supported. Next, the findings revealed that structural empowerment has a significant positive relationship with job performance (β = 0.200; p < 0.05), confirming H4. However, team cohesion demonstrated a significantly negative correlation with job performance (β = −0.267; p < 0.05), contrary to the hypothesized positive effect. Therefore, H5 was not supported.

**Table 6:** Hypothesis testing results.

	Relationship	Std Beta	Std Dev	t-values	p-values	BCI LL	BCI UL	f^2^	Decision
H1	EL > JP	0.855	0.072	11.807	0.000	0.736	0.974	0.471	Supported
H2	EL > SE	0.655	0.029	22.833	0.000	0.602	0.697	0.750	Supported
H3	EL > TC	0.780	0.017	46.577	0.000	0.751	0.806	1.555	Supported
H4	SE > JP	0.200	0.048	3.072	0.000	0.093	0.306	0.059	Supported
H5	TC > JP	−0.267	0.048	5.597	0.000	−0.345	−0.095	0.072	Not Supported
H6	EL > SE > JP	0.131	0.043	3.007	0.000	0.060	0.202	0.0620	Supported
H7	EL > TC > JP	−0.209	0.039	5.307	0.000	−0273	−0.144	0.0125	Supported

Note: EL = empowering leadership; SE = structural empowerment; TC = team cohesion; JP = job performance.

To assess the mediating roles of structural empowerment and team cohesion in the relationship between empowering leadership and job performance, we applied Preacher et al. [105] approach to testing indirect effects. This method states that the confidence interval should not include zero to establish a significant mediating effect. As shown in Table 6, the results indicated a significant impact of empowering leadership on job performance through both structural empowerment (β = 0.313, p < 0.05, LL = 0.060, UL = 0.202) as a positive mediator and team cohesion (β = −0.209, p < 0.1, LL = −0.273, UL = −0.144) as a negative mediator. The confidence intervals of both relationship paths do not straddle zero, verifying H6 and H7. Together, these findings provide empirical support for the dual-path mediation model.

Additionally, the R^2^ values for structural empowerment, team cohesion, and job performance were found to be 0.429, 0.609, and 0.675, respectively. This means that empowering leadership explained 42.9% of the variance in structural empowerment and 60.9% of the variance in team cohesion, whereas empowering leadership, structural empowerment, and team cohesion jointly explained 67.5% of the variance in job performance. As all R^2^ values were above the threshold of 0.26 suggested by Cohen [106], it can be concluded that the model has substantial explanatory power.

Next, according to Hair et al. [107], the effect size (f^2^) statistic evaluates whether the omitted construct has a meaningful effect on the endogenous constructs. It should be calculated by omitting a specific exogenous construct from the model and observing the resulting change in R^2^. Based on Cohen [108] guidelines, f^2^ values of 0.02, 0.15, and 0.35 represent small, medium, and large effects, respectively. Table 6 shows that all direct predictors had substantive effects, with three showing large effects and two showing medium effects. To measure the mediation effect sizes, we referred to Preacher et al. [109] criteria of 0.01, 0.09, and 0.25 representing small, medium, and large effects, respectively. Based on this, the results in Table 6 indicate that the mediators exhibited one small effect and one medium effect.

### 4.4. PLS_Predict_

To assess our model’s predictive performance, we followed the procedure outlined by Shmueli and colleagues and evaluated the out-of-sample predictive power of the model using PLS_predict_ [110]. We performed k-fold cross-validation with k = 9 subgroups to ensure at least 53.6 cases in each holdout sample. In the first stage, we observed that all Q^2^ predict values for all indicators of dependent variables were positive (Table 7). In the second step, because the prediction errors were symmetrically distributed, we used the root mean squared error to assess the degree of prediction error. In this case, the root mean squared error PLS values produced all positive prediction errors (Table 7), thus indicating the level of out-of-sample predictive performance of the model could not predict out in this study. These results indicate that the research model could yield generalizable findings for other data sets and potentially equivalent contexts.

**Table 7:** PLS_Predict_ results.

Item	Q^2^predict	PLS-SEM_RMSE	LM_RMSE	RMSE PLS-LM
JP1	0.312	0.402	0.334	0.068
JP2	0.531	0.491	0.308	0.183
JP3	0.191	0.422	0.4	0.022
JP4	0.21	0.429	0.407	0.022

## 5.0. Discussion

This research contributes to a deeper understanding of the mechanisms that facilitate the effectiveness of empowering leadership, particularly in the education sector. First, applying the SET, we have established that empowering leadership positively influences teachers’ work performance (H1), structural empowerment (H2), and team cohesion (H3). The results of this study are consistent with previous research that highlights the significant impact of empowering leadership on performance [111]. Prior literature also suggests that empowered employees under the influence of empowering leadership develop a sense of ownership of their work, which positively affects their performance [112]. Empowering leadership is an important driver of successful employee empowerment because empowering leaders promote employee autonomy by creating an environment that is conducive to the development of individual potential [58]. Empowering leadership also promotes motivation, which is believed to promote improved employee performance and facilitate the acquisition of work-related skills by encouraging their work roles [113]. This is consistent with SET, where empowering leaders provide support to employees, thereby generating reciprocity in the form of enhanced task performance. Empowering leaders tend to exhibit strong communication, engage in meaningful conversations, and learn from their failures. They also prioritize trust-building, skill development, and the creation of an inclusive workplace, which enhances employee satisfaction and performance as well as organizational resilience, innovation, and long-term success. These findings align with the work of Schaufeli et al. [114], who emphasized that a leader’s flexibility and openness to new ideas enhance employees’ sense of engagement and empowerment.

While we found empirical evidence that structural empowerment enhances job performance (H4), our results surprisingly indicate that team cohesion decreases job performance (H5). This contradicts Gundersen et al. [70] argument that team cohesion boosts performance in decision-making, strategy implementation, and organizational goal attainment. Cohesive teams benefit from higher levels of affinity, trust, satisfaction, and emotional attachment among members [88]. It is likely that there may be different cohesive team dynamics among school teachers, such as an emphasis on group harmony over task performance, which is common in collectivistic cultures like Hong Kong [64].

By delegating authority, involving employees in decision-making, and providing opportunities for skill development, empowering leaders enhance employees’ autonomy, competence, and relatedness, which increases intrinsic motivation and drives higher job performance. At the same time, empowering leadership strengthens structural empowerment by providing access to resources, information, and decision-making authority, enabling employees to utilize these structures effectively and contribute to organizational goals. Additionally, empowering leaders foster team cohesion through collaboration, open communication, and mutual respect, which satisfy relatedness needs and encourage reciprocal exchanges of support and cooperation among team members.

On the other hand, while team cohesiveness is often seen as a driver of improved job performance, this relationship may not hold true in all professional contexts—particularly in education. Among teachers, the link between team cohesiveness and job performance can be weakened or even rendered insignificant due to the nature of their work and individual job characteristics. Teaching is often an autonomous and individualized profession, where performance is typically evaluated based on personal classroom outcomes rather than collective team efforts. As such, high team cohesion may not directly translate into better individual performance, especially if collaboration is not structurally integrated into their daily responsibilities. Additionally, demographic variables such as age, experience level, and educational background can influence how teachers interact within teams. For instance, veteran teachers may prioritize independence over collaboration, while those teaching specialized subjects might have limited opportunities for team-based engagement. In such scenarios, cohesiveness might contribute more to emotional support and collegial satisfaction than to measurable improvements in job performance. These factors suggest that team cohesiveness alone is insufficient to drive performance outcomes unless it is meaningfully linked to shared tasks, coordinated goals, and performance-based exchanges. Therefore, the weak or non-existent relationship between cohesiveness and job performance among teachers highlights the need to consider job-specific demands and demographic influences when evaluating the effectiveness of team dynamics in educational settings. As such, this unexpected finding is worth further exploration.

Notably, the findings validate the significant mediating roles of structural empowerment (H6) and team cohesion (H7) in the effect of empowering leadership on job performance. The importance of structural empowerment as an underlying mechanism primarily arises from the influential and guiding role of empowering leadership. As a positive leadership style, empowering leadership provides structured emotional and cognitive support to employees, enabling them to gain autonomy and decision-making authority in their work. Subsequently, their increased structural empowerment boosts the sense of control over their tasks, making employees feel more capable, productive, and motivated to improve their job performance. However, team cohesion demonstrated a negative mediating effect. In Chinese collectivist settings, excessive team cohesion can lead to conformity pressure, discouraging dissent and creative risk-taking. When cohesion becomes too strong, team members may prioritize maintaining interpersonal harmony over voicing differing opinions or challenging ineffective practices. This dynamic can reduce individual autonomy—ironically contradicting the very purpose of empowering leadership, which seeks to enhance independence and self-driven performance. In other words, the positive influence of empowering leadership on job performance becomes negative when team cohesion is cultivated. This interesting finding implies that while empowering leadership can enhance Hong Kong teachers’ cohesion, such cohesion may impede performance by placing social harmony above productivity. These novel insights add value to the existing literature and lay the groundwork for future research.

## 6.0. Implications and conclusion

### 6.1. Theoretical contributions

This study makes several contributions to the existing literature. First, previous studies have often overlooked the role of context, which is crucial as different settings require different approaches to empowerment. Indeed, much of the extant research has been based primarily on data from mainland China and Western countries, raising concerns of cultural bias in the findings. Thus, this study broadens the scope of research on empowering leadership and job performance, focusing on the cultural context of Hong Kong to reflect the values of non-mainland Chinese. By doing so, we validate the universality of job performance in different cultural environments and shed light on how culture impacts leadership empowerment and job performance, enriching both theoretical and empirical discussions. Moreover, we have addressed a contextual gap in the research on empowering leadership and job performance by examining secondary schools. While prior studies have focused on industries such as commercial industries [15], banking sectors [16], hospitality [17], pharmaceutical [18], manufacturing [19], and construction [20], academic institutions have been largely overlooked. As findings from other industries may not be applicable to secondary schools, we have provided insights specific to the education sector.

Second, this study is among the first to explore the mediating role of structural empowerment in the relationship between empowering leadership and job performance. While earlier research has examined the direct influence of empowering leadership on structural empowerment [49] and its subsequent effect on job performance [63], few studies have investigated the mediation path linking these concepts. By including structural empowerment as a mediating variable, this study answers the call by researchers [77] for more research on this mechanism, offering new insights into how empowering leadership indirectly enhances job performance.

Third, while prior studies have often explored the psychological, motivational, and emotional dimensions of the relationship between empowering leadership and job performance [38], this study takes a behavioral perspective. Specifically, we examined how leader behaviors such as delegating authority [44], expressing confidence in team members [57], and using positive feedback [55] influence team cohesion and, consequently, job performance. Notably, our findings on the negative role of team cohesion challenge existing evidence, opening new pathways for research.

Fourth, notably, the unexpected result of team cohesion’s inability to significantly boost job performance deviates from the prevailing trends observed in previous empirical research, thereby adding a novel dimension to the findings of this study. From the perspective of SET, this unexpected relationship can be understood by examining the nature and quality of exchanges within cohesive teams. High cohesion does not always guarantee effective performance, especially if the cohesion is built on personal affinity rather than task-oriented collaboration. In such cases, strong social bonds may lead to groupthink, complacency, or tolerance of underperformance, as members prioritize harmony over accountability [115].

Finally, this study adds valuable empirical support for the SET and SDT. This theory posits that the relationship between leaders and subordinates is a negotiated process, where reciprocity plays a key role. When organizations offer a resource-rich environment and job empowerment, employees are more likely to reciprocate with higher levels of job performance [116]. Empowering leaders, who foster a trusting work environment and enhance employees’ decision-making abilities, encourage this reciprocal relationship. Employees who feel trusted are more likely to respond with better work outcomes. Additionally, empowering leaders provide employees with decision-making autonomy, information-sharing opportunities, and skill development, which improve their sense of structural empowerment and team cohesion. As a result, employees feel more motivated, optimistic about their work, and driven to enhance productivity. Overall, this study advances the applicability of the SET to a parallel mediation model, supporting the impact of empowering leadership on job performance via the key mechanisms of structural empowerment and team cohesion.

### 6.2. Practical contributions

The findings of this study have important practical implications for secondary schools’ senior management in developing frameworks to enhance teachers’ job performance. First, the positive correlation between empowering leadership and job performance indicates that, in today’s dynamic work environment, it is essential to cultivate school leaders with empowering leadership traits. This highlights the value of leadership training and development programs as a resource to help leaders improve their skills and effectively demonstrate empowering behaviors [45]. Consequently, leaders can focus on skill development and provide the necessary resources and support to help employees grow in their roles. From the perspective of the SET and SDT, when teachers feel empowered and secure, they recognize their responsibility to achieve the school’s goals, thus improving their performance.

Second, due to the direct and mediating positive influence of structural empowerment on job performance, schools should develop strategies to strengthen their structures. For example, creating a psychologically safe and supportive work environment, fostering openness among leaders, and promoting fairness in the reward and punishment system can encourage structural empowerment. Leaders should also work to raise awareness of structural empowerment, grant employees more decision-making power and autonomy, and foster a supportive organizational culture to enhance employees’ sense of empowerment.

Third, considering the direct and mediating negative influence of team cohesion on job performance, we recommend that school leaders practice caution in connecting teams through empowerment. Rather, they should implement strategies that mitigate the negative outcomes of team cohesion. For instance, schools can leverage knowledge sharing [73], schedule regular team seminars, encourage in-depth communication, and promote effective interactions to generate mutual understanding and strengthen team identity without compromising performance. Additionally, making internal reward systems more transparent and equitable can create a harmonious atmosphere that improves overall work performance and supports organizational effectiveness [117].

Fourth, the findings of this study offer several practical implications for school leadership training and policymaking, particularly concerning the balance between teacher autonomy and group cohesion in Hong Kong’s collectivist educational context. School leaders and policymakers should recognize that while team cohesion fosters collaboration, trust, and a sense of belonging, excessive cohesion may inhibit open dialogue, innovation, and individual accountability, especially when group harmony becomes more valued than performance outcomes. Therefore, leadership training programs should focus on developing leaders’ ability to balance empowerment with collective alignment. First, school leadership training should emphasize adaptive empowerment, where leaders gradually delegate authority and decision-making power to teachers based on their readiness, confidence, and task expertise [118]. This approach helps prevent confusion or resistance in highly cohesive teams that may not be accustomed to autonomy. Second, leaders should be trained to encourage constructive dissent and open communication within teams. Techniques such as structured team discussions, rotating leadership roles, or peer mentoring can help teachers express divergent ideas while maintaining mutual respect and cohesion. Third, training programs should equip leaders with cross-cultural communication and conflict management skills, enabling them to navigate the tension between maintaining team harmony and promoting individual initiative—a challenge common in collectivist school cultures [119]. For policymakers, these insights suggest the need to design professional development frameworks that integrate both collaborative leadership and empowerment principles. This could include revising teacher evaluation systems to reward both team contributions and individual creativity, thereby signaling institutional support for balanced leadership practices. Such an approach can enhance both individual job performance and collective school effectiveness, ensuring sustainable improvement in educational outcomes.

### 6.3. Limitations and recommendations for future research

Despite its contributions, this study is not without its limitations. First, our sample was drawn from four secondary schools in Hong Kong, which may limit the generalizability of the findings to other industries and regions. Future research should replicate our framework in a wider range of organizations and cultural contexts to improve the applicability of the results. Second, the cross-sectional design of this study limits the ability to infer causal relationships. Future research should consider a longitudinal or multi-wave design, which would allow for the observation of changes in leadership behavior and employee outcomes over time. Such an approach could help confirm the causal pathways proposed in the model, reduce the risk of common method bias, and provide a clearer understanding of how empowering leadership and its mediating mechanisms evolve and influence job performance in dynamic organizational settings. Third, this study’s examination of job performance is limited, as the framework includes only three predictors and two mediators. Future studies should explore additional variables, such as perceived organizational support, person-job fit, and work engagement, which may directly or indirectly influence job performance. Moreover, the incorporation of moderators like proactive personality could further clarify the contextual impact of empowering leadership on job performance.
